# Ultrafine particles and platelet activation in patients with coronary heart disease – results from a prospective panel study

**DOI:** 10.1186/1743-8977-4-1

**Published:** 2007-01-22

**Authors:** Regina Rückerl, Richard P Phipps, Alexandra Schneider, Mark Frampton, Josef Cyrys, Günther Oberdörster, H Erich Wichmann, Annette Peters

**Affiliations:** 1 Institute of Epidemiology, GSF National Research Centre for Environment and Health, Neuherberg, Germany; 2 Department of Environmental Medicine – Lung Biology and Disease Program, University of Rochester School of Medicine and Dentistry, Rochester, NY, USA; 3 Institute of Epidemiology, GSF National Research Centre for Environment and Health, Neuherberg, Germany; 4 Department of Medicine – Pulmonary and Critical Care Division, University of Rochester School of Medicine and Dentistry, Rochester, NY, USA; 5 Institute of Epidemiology, GSF National Research Centre for Environment and Health, Neuherberg, Germany and WZU – Environmental Science Centre of the University Augsburg, Augsburg, Germany; 6 Department of Environmental Medicine, University of Rochester School of Medicine and Dentistry, Rochester, NY, USA; 7 Institute of Epidemiology, GSF National Research Centre for Environment and Health, Neuherberg, Germany; IBE Chair of Epidemiology, Ludwig-Maximilians-University of Munich, Munich, Germany and Focus-Network Aerosols and Health, GSF National Research Center for Environment and Health, Germany; 8 Institute of Epidemiology, GSF National Research Centre for Environment and Health, Neuherberg, Germany and Focus-Network Aerosols and Health, GSF National Research Center for Environment and Health, Neuherberg, Germany

## Abstract

**Background:**

Epidemiological studies on health effects of air pollution have consistently shown adverse cardiovascular effects. Toxicological studies have provided evidence for thrombogenic effects of particles.

A prospective panel study in a susceptible population was conducted in Erfurt, Germany, to study the effects of daily changes in ambient particles on various blood cells and soluble CD40ligand (sCD40L, also known as CD154), a marker for platelet activation that can cause increased coagulation and inflammation.

Blood cells and plasma sCD40L levels were repeatedly measured in 57 male patients with coronary heart disease (CHD) during winter 2000/2001. Fixed effects linear regression models were applied, adjusting for trend, weekday and meteorological parameters.

Hourly data on ultrafine particles (UFP, number concentration of particles from 0.01 to 0.1 μm), mass concentration of particles less than 10 and 2.5 μm in diameter (PM_10_, PM_2.5_), accumulation mode particle counts (AP, 0.1–1.0 μm), elemental and organic carbon, gaseous pollutants and meteorological data were collected at central monitoring sites.

**Results:**

An immediate increase in plasma sCD40L was found in association with UFP and AP (% change from geometric mean: 7.1; CI: [0.1, 14.5] and 6.9; CI: [0.5, 13.8], respectively). Platelet counts decreased in association with UFP showing an immediate, a three days delayed (lag 3) and a 5-day average response (% change from the mean: -1.8; CI: [-3.4,-0.2]; -2.4; CI: [-4.5,-0.3] and -2.2; CI: [-4.0,-0.3] respectively).

**Conclusion:**

The increased plasma sCD40L levels support the hypothesis that higher levels of ambient air pollution lead to an inflammatory response in patients with CHD thus providing a possible explanation for the observed association between air pollution and cardiovascular morbidity and mortality in susceptible parts of the population.

## Background

Particulate air pollution has consistently been associated with adverse cardiovascular events [1-5], however the underlying pathophysiological mechanisms remain unclear.

Different pathways, including systemic inflammation, an imbalance in coagulation factors and effects on the autonomic nervous system have been postulated [6].

Recent toxicological studies have demonstrated a pro-thrombogenic effect of diesel exhaust particles in hamsters, as well as an activation of platelets [7], thus providing one possible explanation for the observed effects. Studies on rats additionally showed evidence for thrombus formation after the deposition of ultrafine particles (UFP, number concentration of particles from 0.01 to 0.1 μm in diameter,) in the respiratory tract [8] while a study in mice indicated a procoagulant effect, but no inflammation after intra-arterial infusion of UFP [9]. Nemmar et al. [10] demonstrated in hamster models that ultrafine polystyrene particles can modulate thrombus formation and Suwa et al. [11] observed a systemic inflammatory response and a progression of the atherosclerotic process in hyperlipidemic rabbits in association with PM_10 _(mass concentration of particles < 10 μm in diameter).

We previously observed a delayed increase in levels of C-reactive protein (CRP) and intercellular adhesion molecule 1 (ICAM-1) above the 90^th ^percentile for an increase in accumulation mode particles (AP, 0.1–1.0  μm), UFP and PM_10 _in the same patients with coronary heart disease (CHD) we report about here [12]. Clotting factors, however, did not reveal a clear picture. While prothrombin fragment 1+2 increased with a delay of four days in association with ambient particles, no consistent results were found for fibrinogen. For FVII clear and consistent negative associations were observed [12].

In addition to the above mentioned markers, we analysed now the effect of particulate air pollution on blood cells (platelets, leukocytes, erythrocytes, haemoglobin) and soluble CD40L (sCD40L). CD40L, a glycoprotein released from activated platelets [13], promotes the process of thrombus formation in vivo [14]. Moreover, both surface bound and released CD40L is proinflammatory, prothrombotic and proatherogenic [15]. CD40L can strongly activate CD40 bearing cells such as white blood cells, endothelial cells and platelets themselves [16,13].

The aim of this analysis was to study the effects of ambient air pollution on blood cells and sCD40L in a susceptible population in view of previous results. We hypothesised that sCD40L, as well as blood cell counts, would increase in tandem with increased ambient particles. For the analysis, repeated measurements of sCD40L, platelets, leukocytes, erythrocytes and haemoglobin were related to concurrent levels of air pollution in a panel of male patients with CHD.

## Materials and methods

### Study design

In a prospective panel study conducted between October 15^th ^2000 and April 27^th ^2001 in Erfurt, Germany as part of the University of Rochester (Rochester, NY, USA) Particle Center Study current non-smokers, aged 50 years or older, with physician-diagnosed CHD were recruited through a local cardiologist. Patients who had pacemakers, recent (less than three months ago) myocardial infarction, bypass-surgery or balloon dilatation, type 1 diabetes or were on anti-coagulation therapy (except for anti-platelet agents) were not included. From each subject a signed informed consent was obtained.

Sixty-one male patients with CHD were scheduled for 12 subsequent clinical examinations over a period of six months. Each clinical visit included a short interview and the withdrawal of a blood sample under non-coagulating conditions using citrate tubes. Blood cells from whole blood were counted immediately following each visit. For the baseline characterisation a detailed questionnaire was administered at the first visit, regarding health status, pulmonary and cardiac symptoms, medication intake and smoking history.

All methods used in the study were conducted according to Standard Operating Procedures (SOP) and were tested beforehand in a two-week pilot study. The study protocol was approved by the German Ethics Commission "Bayerische Landesaerztekammer".

### Study subjects

Out of the initial 61 patients 57 remained for the analyses. One patient refused to participate; two were excluded due to diseases that led to a change in blood markers (leukaemia, lymphoma) and the fourth patient because of constantly elevated levels of leukocytes and erythrocytes, indicating an unknown underlying disease. Most patients (n = 55) participated in all of the 12 scheduled visits, while two took part in only nine and eight examinations respectively. For comparability reasons we included the same blood samples that were included in the previous analyses [12]. If patients reported an acute infection and/or surgery during the two weeks prior to the examination or if the nurses saw signs of an acute infection the blood samples were excluded from the analysis (46 blood samples of 19 different patients). Finally, not all patients were able to give the scheduled amount of blood at each visit. Therefore, between 539 and 579 blood samples remained for analysis depending on the marker/blood cell.

### Clinical measurements

For each patient the clinical visits were scheduled on the same weekday (Monday to Friday) and time (8:00 am to 5:00 pm) once every two weeks.

At each visit a sample of 4 × 1.0 ml citrate plasma was drawn (Becton Dickinson, NJ, USA) using 20 or 21-gauge needles. Samples were cooled down and centrifuged at 2500 g for 20 minutes at a temperature of 4°C and the cell free aliquots stored at -20°C, immediately.

After the collection was completed the blood samples were shipped to the USA on dry ice for sCD40L determination. sCD40L levels in blood plasma were determined with an ELISA for sCD40L. For the analyses of sCD40L all samples were processed the same and appropriately diluted so that at least one value fell on the standard curve. A 96-well microtiter plate was coated with 3 μg per ml of mouse anti-human sCD40L antibody (gift of Dr. Marilyn Kerry, Boeringer-Ingelheim, CT, USA) and allowed to incubate overnight at room temperature. A blocking buffer consisting of PBS (phosphate buffered normal saline) -1% BSA (bovine serum albumin) and 0.1% NaN_3 _was added to the plate and incubated for one hour. The plate was then washed three times using a washing buffer consisting of PBS containing 0.05% Tween 20 and 0.1% NaN_3_. Recombinant human sCD40L standards (Bender Medsystems, Vienna, Austria) were added to each plate using serial dilutions in blocking buffer to create a standard curve of 6.25 to 400 pg/ml. Samples were diluted as needed with blocking buffer and added to the plate. The plates were incubated for two hours and then washed three times with washing buffer. Biotinylated mouse monoclonal anti-human CD40L antibody (Ancell, Bayport, MN, USA) was added to each well (2 μg per ml in blocking buffer) and incubated for two hours. The plates were washed three times with washing buffer. A 1 to 1000 dilution of streptavidin-alkaline phosphatase (Bio-Rad Laboratories, Hercules, CA, USA) was added to each well and allowed to incubate 30 minutes before washing three times with washing buffer. The ELISA plates were then developed using an alkaline phosphatase substrate kit (Bio-Rad Laboratories, Hercules, CA, USA) as per manufacturer's directions for use. After incubating 20–30 minutes in the dark, the ELISA plates were promptly read using a microplate reader (Bio-Rad, Hercules, CA, USA) at wavelength of A405 with a reference of A610 [17].

The haemograms of the specimens including platelets, leucocytes, erythrocytes and haemoglobin were obtained by an Abbott Cell-Dyn 1300 cell counter (Abbott, Ill, USA) from whole blood immediately following each visit.

### Air pollution monitoring

The concentrations of different ambient air pollutants were measured at a fixed monitoring site representing urban background levels. The measurement site was located approximately one kilometre south of the Erfurt city centre and 30 m from the nearest major road. The measurement site was established especially for carrying out epidemiological studies, [18] and all measurements were conducted according to the SOP developed within the framework of previous studies [19].

Continuous UFP counts, accumulation mode particle counts and fine particle mass (PM_2.5_, particles < 2.5 μm diameter) were measured with a Mobile Aerosol Spectrometer (MAS). The MAS, described previously [20] consisted of two different commercially available instruments covering different size ranges. Particles in the size range from 0.01 μm to 0.5 μm were measured using a differential mobility particle sizer (DMPS). Particles in the size range from 0.1 μm up to 2.5 μm were classified by an optical laser aerosol spectrometer (LAS).

Mass concentration of PM_10 _data was collected by the tapered element oscillating microbalance method (TEOM 1400a) and continuous data on elemental and organic carbon (EC, OC) were measured with an ambient carbon monitor (ACM 5400) (Rupprecht & Patashnick, Co., Inc., Albany, NY, USA). Data on meteorological variables for this period as well as concentrations of gaseous air pollutants were collected from existing networks.

Missing values in the ambient particle pollutants UFP and PM_2.5 _between 20^th ^of January and 13^th ^of February 2001 and one missing day (31^st ^of March 2001) in the meteorological variables of temperature and relative humidity were either imputed by a linear regression model (UFP) or replaced based on corrected concurrent measurements with other devices (PM_2.5_, temperature, relative humidity). The squared multiple correlation for the UFP regression models was 0.96.

### Statistical analyses

For each person five individual periods covering 24-hour averages of pollutants were calculated if more than 2/3 of the hourly measurements were available for this period. We used the 24 hours immediately preceding each clinical visit (lag 0), the period 24 to 47 hours (lag1) up to day 5 prior to the visit (lags 2 to 4) and 5-day running means to measure cumulative effects.

Generalized additive models including pollutant and confounder variables were used for fixed effects regression with individual intercepts for each patient. Long-term time trend, weekday of the visit and the meteorological parameters air temperature, relative humidity and barometric pressure, each with lag 0 to lag 3, were considered as potential confounders. As the visits took place in two week intervals, we assumed that no adjustment was necessary for autocorrelation in the pollution data and most of the blood markers. For the erythrocytes, which have a longer half life, sensitivity analyses were conducted to check for autocorrelation. Eight patients who showed an indication for autocorrelation for erythrocytes were excluded and the reduced dataset was re-analysed.

sCD40L needed to be log-transformed to fulfil the model assumption of residual normality. To explore the shape of the association between confounders and blood markers, non-parametric smooth functions based on locally weighted least squares were applied. Model fit was rated based on the Akaike Information Criterion (AIC). In the final model, non-parametric smooth functions were replaced by appropriate polynomials (degree 2 or 3) or natural splines with similar number of degrees of freedom. Parametric functions were used to avoid an underestimation of the standard errors which can occur if concurvity is present in the data, as is often the case in air pollution studies [21].

Effect estimates are presented as percent change of the mean or geometric mean together with 95 percent confidence intervals (95%CI) based on an increase in air pollution concentrations from the 1^st ^to the 3^rd ^quartile (interquartile range, IQR). Data were analyzed using the statistical package SAS Version 8.2 (SAS Institute Inc.) and S-Plus Version 6.0 (Math Soft Inc.). A subgroup analysis was conducted comparing patients who were on lipid-lowering drugs to those who were not.

## Results

### Patient characteristics

The patient characteristics are summarized in Table 1. The study population comprised 57 non-smoking men with CHD, between 51 and 76 years. Although all patients were current non-smokers, three quarters of the patients were former smokers. The majority took acetylsalicylic acid, half of them were on lipid-lowering medication like statins and three of the patients used platelet aggregation inhibitors.

**Table 1:** Characteristics of the study population in Erfurt, Germany, winter 2000/2001

		**Mean (SD^‡^)**
**Age [years]**		66 (6.0)
BMI* [kg/m^2^]		28 (3.4)
		
		No. (%)
**History of**		
	Coronary heart disease	57 (100)
	Angina pectoris	40 (68)
	Myocardial infarction	43 (75)
	Bypass surgery/balloon dilatation	49 (86)
	Stroke	3 (5)
	Diabetes mellitus	13 (23)
	Hypertension	40 (70)
	Chronic bronchitis	2 (4)
	COPD^†^	5 (9)
	Hay fever	2 (4)
	Chronic kidney disease	6 (11)
		
**Smoking**		
	Never smoker	15 (26)
	Ex-smoker	42 (74)
		
**Medication use**	Anti-hyperlipidemic medication	29 (51)
	Platelet aggregation inhibitors	
	Acetylsalicylic acid	52 (91)
	Thienopyridines	3 (5)

* BMI, Body mass index

† COPD, Chronic obstructive pulmonary disease

‡ SD, standard deviation

### Air pollutants

The distributions of the 24-hour average concentrations of the particulate and gaseous pollutants, as well as of the meteorological data are shown in Table 2.

**Table 2:** 24-h-averages of air pollutants and meteorological variables between October 2000 and April 2001

**Variable**	**N**	**Mean (± SD^#^)**	**Min.**	**25%**	**Median**	**75%**	**Max.**	**IQR^††^**	**IQR^†† ^5-day average**
UFP* [n/cm^3^]	196	12602 (± 6455)	2542	7326	11444	17332	34294	10005	6821
AP* [n/cm^3^]	167	1593 (± 1034)	328	821	1238	2120	4908	1299	1127
PM_2.5_*[μg/m^3^]	197	20.0 (± 15.0)	2.6	9.7	14.9	26.1	83.7	16.4	12.2
PM_10_*[μg/m^3^]	154	20.0 (± 13.0)	5.4	10.8	15.6	26.0	74.5	15.2	12.8
**NO^†^[μg/m^3^]**	196	24.4 (± 27.7)	4.0	6.8	12.3	30.3	137.6	23.5	21.0
**Air temperature^‡^[°C]**	198	4.1 (± 4.8)	-10.4	0.5	4.4	7.9	13.2	7.4	5.9
**Barometric pressure [hPa]**	198	973.4 (± 9.7)	949.5	966.3	972.9	980.0	995.7	13.6	10.8
**Relative humidity**^§ ^**[%]**	198	83.5 (± 8.8)	55.8	78.9	84.3	88.8	100.0	10.0	9.1

* UFP, ultrafine particles, number concentration of particles with a size range of 0.01 to 0.1 μm in diameter

13% of the hourly measurements were imputed,

AP, Accumulation mode particles, particles with a size range of 0.1 to 1.0 μm,

PM_2.5_, mass concentration of particles less than 2.5 μm in diameter

15% of the hourly measurements were imputed

PM_10_, mass concentration of particles less than 10 μm in diameter

† NO, Nitrogen monoxide;

‡ Temperature: 0.5% of the hourly measurements were imputed

^§^Relative humidity: 0.5% of the hourly measurements were imputed

# SD, standard deviation

†† IQR, inter quartile range

Due to the different measurement methods mean values for PM_10 _and PM_2.5 _are almost the same. The TEOM device, that measured PM_10_, is kept at a constant temperature of 50°C, which can lead to partial or total volatilization of light volatile compounds, producing a recorded mass below that of the true aerosol mass in ambient air [22,23]. The MAS, on the other hand, provides online measurements, so no volatilization is to be expected.

**Figure 1:**
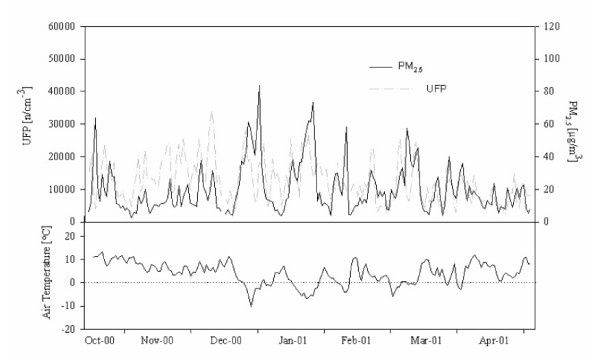
**Time series of number concentrations of particles sized 0.01 to 0.1 μm (ultra fine particles, UFP) and mass concentrations of particles less than 2.5 μm in diameter (PM_2.5_) together with air temperature in Erfurt, Germany between October 2000 and April 2001**. UFP: ultrafine particles (number concentration of particles with a size range of 0.01 to 0.1 μm); PM_2.5_: mass concentration of particles less than 2.5 μm in diameter.

PM_10_, PM_2.5 _and AP were highly correlated (r = 0.86 to 0.91), whereas UFP were only moderately correlated with PM_10 _and PM_2.5 _(r = 0.57 and 0.41 respectively) (Figure 1). NO and UFP also showed a high correlation (r = 0.83).

### Blood parameters

Table 3 shows the values of sCD40L, platelets, leukocytes, erythrocytes and haemoglobin in the analysed blood samples. Erythrocytes and haemoglobin were highly correlated (r = 0.74) and leukocytes and platelets showed a moderate correlation (r = 0.52). No correlation was seen between sCD40L and all of the analysed blood cells (r = -0.02 to -0.32).

**Table 3:** Levels of sCD40L, platelets, leukocytes, erythrocytes and haemoglobin for 57 patients with coronary heart disease

	**N**	**Mean**	SD*	**Min**	**Median**	**Max**
**sCD40L^† ^[pg/ml]**	539	5246	4481	115	4129	44260
**Platelets [10^3^/μl]**	578	185	65	41	175	603
**Leukocytes [10^3^/μl]**	579	6.8	1.7	2.8	6.7	14.5
**Erythrocytes [10^6^/μl]**	578	4.6	0.4	3.4	4.6	6.0
**Haemoglobin [g/dl]**	579	14.1	1.0	8.7	14.2	17

*SD: Standard deviation of all values

^†^sCD40L, soluble CD40 Ligand

### Regression results

#### sCD40L, platelets and leukocytes

The results of the regression for sCD40L, platelets and leukocytes are given in Table 4. Linear regression suggested an increase in sCD40L in association with 24-h average ambient particles before the examination. The effects were significant for UFP and AP. The effect of the concentrations of UFP was supported by the result for NO, a gaseous marker for UFP, which also showed an increase with lag 0 that was borderline significant (%change from geometric mean: 3.2; CI: [-0.2; 6.8], p < 0.07) (data not shown). Additionally, a decrease with lag 3 was found that was limited to UFP.

**Table 4:** Association between air pollution and blood parameters; 57 male patients with coronary heart disease

	**sCD40L [pg/ml]**		**Platelets [10^3^/μl]**		**Leucocytes [10^3^/μl]**	
	**%change GM^$^**	**95% CI^$^**	**%change mean**	**95% CI**	**%change mean**	**95% CI**

**UFP^§^**						
**lag0**	7.1*	0.1; 14.5	-1.8*	-3.4; -0.2	-2.4*	-4.5; -0.2
**lag1**	0.3	-6.6; 8.6	-1.1	-2.9; 0.6	-2.1^†^	-4.4; 0.2
**lag2**	0.6	-5.9; 8.6	-1.0	-2.9; 0.8	0.2	-2.4; 2.8
**lag3**	-8.5*	-15.8; -0.5	-2.4*	-4.5; -0.3	-1.5	-4.4; 1.4
**5-day-average**	-0.7	-7.6; 6.8	-2.2*	-4.0; -0.3	-1.6	-4.1; 0.8
						
**AP^§^**						
**lag0**	6.9*	0.5; 13.8	-1.0	-2.5; 0.5	-1.9*	-3.8; -0.1
**lag1**	-1.1	-8.0; 6.4	-0.4	-2.1; 1.6	-0.6	-2.9; 1.6
**lag2**	-4.9	-11.9; 2.7	0.8	-1.0; 2.4	-0.6	-3.2; 2.0
**lag3**	-3.8	-10.3; 3.2	0.0	-1.8; 1.7	-2.3^†^	-4.6; 0.1
**5-day-average**	-1.3	-9.9; 8.1	-0.9	-3.0; 1.3	-2.7^†^	-5.5; 0.1
						
**PM_2.5_^§^**						
**lag0**	1.5	-4.0; 7.3	-0.6	-1.9; 0.7	-1.6*	-3.2; 0.0
**lag1**	0.2	-5.4; 6.2	0.1	-1.3; 1.5	-0.4	-2.2; 1.4
**lag2**	-2.6	-8.0; 3.1	0.5	-0.9; 1.9	-0.2	-2.1; 1.7
**lag3**	0.5	-3.9; 5.0	0.2	-1.1; 1.5	-0.8	-2.4; 0.7
**5-day-average**	0.2	-5.4; 6.2	-0.4	-1.9; 1.2	-1.6	-3.5; 0.3
						
**PM_10_^§^**						
**lag0**	1.6	-3.5; 7.0	-0.4	-1.9; 1.0	-1.1	-2.8; 0.7
**lag1**	1.1	-5.4; 7.9	0.4	-1.4; 2.3	-0.5	-2.6; 1.5
**lag2**	-3.5	-8.9; 2.2	0.5	-1.4; 2.3	0.1	-2.1; 2.4
**lag3**	-1.4	-6.0; 3.4	-0.1	-1.6; 1.4	-0.7	-2.6; 1.2
**5-day-average**	-1.2	-7.8; 5.8	0.0	2.1; 0.0	-1.1	-3.6; 1.4

*p < 0.05; ^†^p < 0.10

^§ ^UFP: ultrafine particles (number concentration of particles with a size range of 0.01 to 0.1 μm);

AP: accumulation mode particles (particles with a size range of 0.1 to 1.0 μm); PM_2.5_: mass concentration of particles less than 2.5 μm in diameter; PM_10_: mass concentration of particles less than 10 μm in diameter;

^$^CI: confidence interval, GM: geometric mean

For platelets, significant results were limited to UFP showing an immediate and a three day delayed decrease as well as a negative association with the 5-day average concentration. The linear regression of leukocytes showed consistently negative associations for UFP, AP and PM_2.5_, with lag 0 and in addition borderline significant effects for AP with lag 3 and the 5-day average.

**Figure 2:**
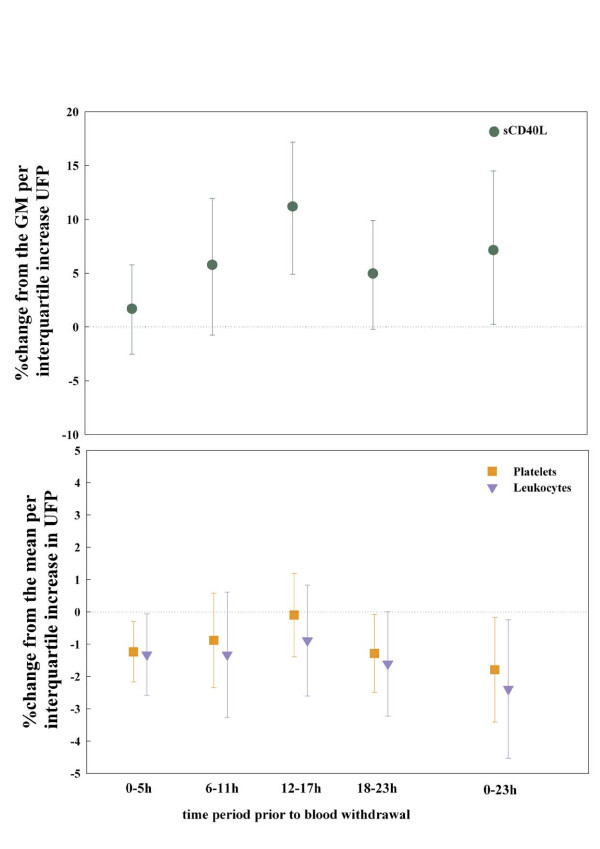
**Association between UFP and sCD40L, platelets and leukocytes, 6-hour periods and lag 0 (0–23 hours)**. sCD40L: soluble CD40Ligand. UFP: ultrafine particles (number concentration of particles with a size range of 0.01 to 0.1 μm).

To further investigate the immediate effects, we split the 24 hours into four six hour periods (0–5 h, 6–11 h, 12–17 h and 18–23 h) and analysed the results for UFP. While the effect for sCD40L was most prominent for the exposure time period 12 to 17 hours prior to the blood withdrawal, platelets and leukocytes showed an immediate decrease in the first six hours and a delayed one between 18 to 23 hours (Figure 2).

#### Erythrocytes and haemoglobin

Erythrocytes and haemoglobin, in contrast, were mainly associated with PM_2.5 _and PM_10 _showing negative associations for the 2 day-lagged concentrations and 5-day averages. Erythrocytes showed additional associations with PM_10 _at lags 1 and 3 and with UFP for lag 3 and the 5-day average exposure (Table 5).

**Table 5:** Association between air pollution and blood parameters; 57 male patients with coronary heart disease

	**Erythrocytes [10^6^/μl]**		**Haemoglobin [g/dl]**	
	**%change mean**	**95% CI^$^**	**%change mean**	**95% CI**

**UFP^§^**				
**lag0**	-0.2	-0.7; 0.2	-0.4	-1.1; 0.2
**lag1**	-0.3	-0.8; 0.2	-0.4	-1.1; 0.3
**lag2**	0.0	-0.6; 0.5	0.0	-0.8; 0.7
**lag3**	-0.6*	-1.3; 0.0	-0.3	-1.1; 0.5
**5-day-average**	-0.5^†^	-1.0; 0.1	-0.4	-1.1; 0.3
				
**AP^§^**				
**lag0**	-0.1	-0.5; 0.3	-0.2	-0.7; 0.4
**lag1**	-0.4	-0.9; 0.2	-0.3	-1.0; 0.4
**lag2**	-0.4	-0.9; 0.2	-0.1	-0.9; 0.7
**lag3**	-0.4	-0.6; 0.3	-0.1	-0.8; 0.6
**5-day-average**	-0.4	-1.0; 0.2	-0.2	-1.1; 0.6
				
**PM_2.5_^§^**				
**lag0**	-0.1	-0.5; 0.3	0.0	-0.6; 0.5
**lag1**	-0.3	-0.7; 0.2	-0.2	-0.8; 0.3
**lag2**	-0.4*	-0.8; 0.0	-0.5*	-1.1; 0.0
**lag3**	-0.2	-0.5; 0.1	-0.2	-0.7; 0.2
**5-day-average**	-0.4^†^	-0.8; 0.0	-0.5^†^	-1.0; 0.1
				
**PM_10_^§^**				
**lag0**	0.0	-0.4; 0.5	-0.1	-0.7; 0.6
**lag1**	-0.4^†^	-1.0; 0.1	-0.4	-1.2; 0.3
**lag2**	-0.7*	-1.2; -0.2	-0.7^†^	-1.3; 0.0
**lag3**	-0.4*	-0.8; 0.0	-0.3	-0.9; 0.2
**5-day-average**	-0.6*	-1.2; -0.1	-0.7^†^	-1.5; 0.1

*p < 0.05

^† ^p < 0.10

^§ ^UFP: ultrafine particles (number concentration of particles with a size range of 0.01 to 0.1 μm);

AP: accumulation mode particles (particles with a size range of 0.1 to 1.0 μm); PM_2.5_: mass concentration of particles less than 2.5 μm in diameter; PM_10_: mass concentration of particles less than 10 μm in diameter;

^$^CI: confidence interval

#### Sensitivity analyses

Excluding eight patients whose data indicated possible autocorrelation did not change the results for erythrocytes significantly. Except for slightly larger confidence intervals, most probably due to the reduced number of data points, no major changes were found when comparing the whole dataset to the reduced one. The estimate for PM_10 _with lag2, for example showed a % change from the mean of -0.8, 95% CI: [-1.3; -0.2].

#### Subgroup analyses

We found no difference in the results for patients who were on lipid-lowering drugs (mainly statins) compared to those who were not (data not shown). As more than 90% of our patients used platelet aggregation inhibitors (Table 1) a subgroup analysis for those with and without intake could not be conducted.

## Discussion

### Summary

Our findings support the concept that sCD40L levels in plasma increase in association with elevated ambient air particle concentrations 24 hours before the examination. The effects were limited to number concentrations of UFP and AP, dominated by particles below 500 nm. Platelets and leukocyte counts decreased in association with an increase in UFP and AP shortly before the examination. For erythrocytes and haemoglobin the main findings were delayed decreases in association with PM_2.5 _and PM_10_.

### sCD40L

CD40L, a trimeric transmembrane protein of the tumor necrosis family, was originally identified on cells of the immune system [24]. It binds to CD40 on cells such as endothelial cells, monocytes and dendritic cells [25]. CD40L is also stored in platelets and translocated to the platelet surface upon stimulation within seconds after activation in vitro and in vivo [14,13]. Surface expressed CD40L is then cleaved and/or released from the platelet over a period of minutes to hours, subsequently generating a soluble fragment, soluble CD40L (sCD40L) [13]. sCD40L can promote inflammatory or thrombotic response by causing further platelet activation [13,26]. This can occur through binding to platelet CD40 [14] or to the integrin [15]. More than 95% of the circulating CD40L exists in platelets [27,13]. André et al. [28] therefore deduced that platelet stimulatory events must be considered in the biological and pathological context of CD40L function. Henn et al. [14] concluded from their study that the generation of inflammatory signals by platelets may occur following acute mechanical damage of the endothelium, an infection of the vascular system, and also in the pathogenesis of atherosclerosis and vascular infarction. Our results indicate an immediate increase in sCD40L in association with increased levels of UFP. A possible explanation is that UFP act as a stimulant for platelets, leading to their release of CD40L into the circulation. According to Geiser et al. [29] particles rapidly translocate into the circulation. Also Nemmar et al. [30] were able to demonstrate that inhaled particles can leave the lungs and enter circulation. However, the circumstances under which particles translocate have to be elucidated and there are other studies that report no translocation [31] or that translocation might depend on lung permeability [32]. Moreover, studies showed increased thrombus formation, an early response presumably due to direct translocation of particles and a 24-hours delayed response due to inflammation in the alveolar region [7,10]. Berry et al. [33] found nanoparticles in blood platelets that had been instilled into rat lungs beforehand. We hypothesise that translocated particles might be responsible for the observed positive association between UFP and the levels of sCD40L.

So far, only a few studies regarding the effect of air pollution particles on the CD40/CD40L pathway have been published [34,35]. In accordance with our results these studies indicate a positive association between ambient particles and sCD40L/CD40 despite the different study design.

### Platelets

**Figure 3:**
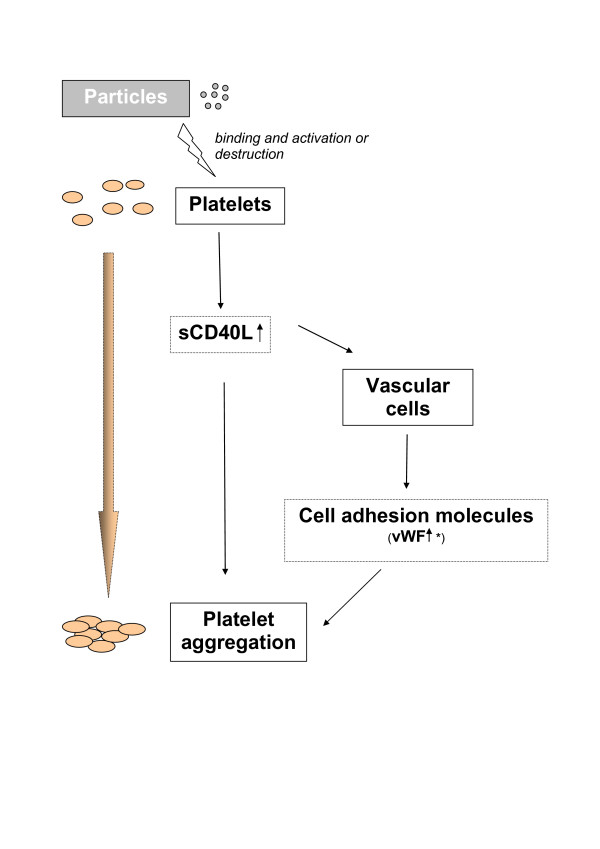
**Schematic representation of the hypothesised mechanisms involved in direct vascular effects of particles or their translocated components**. * see Reference [12]. sCD40L: soluble CD40Ligand. vWF: von Willebrand factor.

Many studies have been published on the association of platelets and ambient air pollution, but the results are not consistent [36-41]. Considering our results of increased plasma levels of sCD40L, the decrease in platelets could be the result of platelet aggregation, either due to a direct activation of the platelets or via an increase in adhesion molecules (Figure 3). Inwald et al. [42] showed that platelets can be activated through ligation of constitutively expressed CD40L. They hypothesised that platelet-derived CD40L may have the capacity to stimulate resting platelets by binding to CD40 during direct cell-cell contact or via release of sCD40L. sCD40L has been shown to initiate inflammatory and thrombotic response by causing further platelet activation, or, in high shear settings, by enhancing the formation of homotypic aggregates [13]. We observed an increase in plasma levels of sCD40L at the same day, and an equally large and significant reduction in sCD40L 72 hours later, while levels of platelets showed a consistent decrease. This decrease in platelet count could be due to changes in platelet activation, which allow them to bind to other tissues such as lung, spleen and vascular cells. Such an activation is expected to cause the platelets to release some of their stores of sCD40L, which could lead to the increased detection of plasma sCD40L. The subsequent decrease in plasma sCD40L levels might be caused by the activation of cells (caused in part by platelet release of sCD40L and other proinflammatory mediators) that now express heightened levels of CD40. Vascular cells, B lymphocytes and fibroblasts can increase their surface expression of CD40 after activation [43,44]. Increases in the cellular expression of CD40 would be predicted to cause a lowering of blood sCD40L.

It has also been shown that platelet CD40L can activate vascular cells, resulting in a production of proinflammatory cytokines and cell adhesion molecules [15,14]. vWF, for example, an important player in the coagulation of platelets, bridges the gap between platelets and collagen, which is exposed if endothelial cells are damaged [45]. In previous analyses of the same panel we found a significant increase in vWf in association with AP, UFP and PM_10 _[12]. An increase in vWf might be another explanation for the observed decrease in platelets.

Other mechanisms are also conceivable. For example, particles could lead to a damage of endothelial cells, with a subsequent adhesion and aggregation of platelets to the damaged part of the vessel wall. Seaton et al. [41] attributed the decrease in platelets to an early consumption coagulopathy secondary to a change in the adhesive properties of erythrocytes.

### Leukocytes

In addition to the decreased levels of platelets we observed a decrease in leukocytes, following the same time pattern. Studies have linked particulate exposure to the level of leukocytes [41,36,39,11,46], however results are contradictory and different hypotheses regarding the underlying mechanisms have been proposed. An increase in leukocytes in association with air pollution is usually interpreted as an indication for an increased inflammatory response. A decrease in leukocytes on the other hand is more difficult to explain and the explanations suggested here can only be hypotheses. Similar to platelets, the low levels in leukocytes we found in peripheral blood in association with an increase in particles, might be a consequence of the increased levels of adhesion molecules. Adhesion molecules like E-selectin and ICAM-1 mediate the contact between circulating leukocytes and endothelial cells. This way, leukocytes can leave the blood stream and enter the subendothelial space [47-49]. Increased levels of adhesion molecules in association with higher levels of air pollution have been shown previously [38,12] supporting the hypotheses above. However, the immediate decrease in blood cells is not in line with the more delayed increase in adhesion molecules.

Ghio et al. [39] found that levels of blood leukocytes decreased in association with CAPs, while levels in bronchial lavage increased in healthy volunteers after exposure to CAPs [50]. They attributed the decrease in peripheral blood to an influx of leukocytes into the lower respiratory tract following exposure to ambient particles.

A third possibility is that the decrease in leukocytes in peripheral blood is the result of a prolonged or delayed transit of leukocytes through the lungs following particle induced vasoconstriction. Pietropaoli et al. [51] showed that inhalation of carbon UFP for two hours reduced the pulmonary diffusing capacity for carbon monoxide in healthy subjects. Frampton et al. [52] reported that similar exposures to UFP reduced the percentage of eosinophils and basophils in peripheral blood, and reduced expression of adhesion molecules on peripheral blood monocytes and granulocytes. Similar results were found in a study on rats, which showed vasoconstriction of small pulmonary arteries after short term exposure to CAPs [53]. These findings support the hypothesis that UFP cause pulmonary vasoconstriction.

### Erythrocytes and Haemoglobin

Regarding erythrocytes and haemoglobin our results are in accordance with those of Seaton et al. [41] who found significant decreases in erythrocytes and haemoglobin in association with personal exposure to PM_10 _as well as with city centre measured PM_10_. They found this decrease after a 3-day average exposure to PM_10_, which fits our data showing significant effects mainly for lag 2 and 3 and the 5-day average exposure. Seaton et al. suggested that after an alteration of adhesive properties of passing erythrocytes they might adhere to the systemic capillaries and therefore not be measurable in peripheral blood anymore.

### Strengths and limitations

As there is no standard way determining sCD40L, several methods have been used in previous studies and it has been shown that the type of anti-coagulant and whether plasma or serum is used may influence the sCD40L levels [27]. However, in our study all samples were analysed at the same time and under the same conditions. The assay used for this study has been validated and a comparison to a kit by Bender Biomedical (Bender Biomedical Systems, San Bruno, CA, USA) showed very similar results [17]. Moreover, the change of the sCD40L level within one patient was used for analysis, rather than the absolute level, and therefore the assay itself cannot have influenced the results.

Some of the measured biomarkers, for example leukocytes, are influenced easily by health-related events such as acute infection or surgery [36]. We therefore carefully excluded the blood samples where the level of sCD40L and blood cells potentially could have been strongly influenced by another source than air pollution, like infection or surgery. However, no samples were excluded that revealed especially high or low levels of the respective biomarker, if no plausible reason was apparent. We observed the associations for changes of the population mean of the respective markers. In contrast to earlier analyses, for example for CRP [54], we did not observe only a shift in the upper tail of the distribution. This indicates that the effects of ambient air pollution may occur within the physiological range of the biomarkers.

We are aware of the fact that leukocyte blood count is not as significant a measure of the changes in blood cells as a differential blood cell analysis. As we do not have a differentiation, we cannot identify which type of leukocyte was responsible for the observed changes.

A variety of pollutants have been used for the analyses, as different pollutants may point towards different properties of the aerosol, and also represent different sources of air pollution. By testing several outcomes and a set of air pollutants, the possibility that some associations might have been observed by chance cannot be excluded. As the air pollution parameters are correlated, we considered especially consistent patterns in the data as actual effects. Moreover, thorough confounder adjustment for meteorological variables was done to rule out the possibility that the detected associations resulted from meteorological influences or seasonal differences.

Only one central measurement site was used for the collection of ambient air pollution. The spatial representativeness of this site has been analyzed in detail previously by Cyrys et al [55] who measured sulphate and PM_10 _levels simultaneously at three additional monitoring sites in the Erfurt area. The relatively high inter-site correlation between the monitoring stations (0.69 – 0.98) indicated that regional episodes of sulphates and PM_10 _in Erfurt can be identified using one fixed monitoring site and that our site was generally representative for the urban background level of air pollution within Erfurt. Erfurt is a small city with one air mass confined by a mountain ridge on three sides and high rises on the fourth side. As most of the participants of our panel were already retired, we assumed that they spent the greater part of their day within the vicinity of their residence within the city of Erfurt.

Janssen et al [56] showed in two European cities that ambient, indoor and personal concentrations of PM_2.5 _were highly correlated. Ebelt et al. [57] also demonstrated that the component of personal exposure due to outdoor particles and ambient concentrations were highly correlated, with a Pearson correlation coefficient of 0.81 for PM_2.5_, of 0.71 for PM_10 _and of 0.73 for the coarse fraction (PM_10 _– PM_2.5_). They concluded that their results add support for the use of ambient monitoring data in time series analyses. Cyrys et al [58], who compared the relationship of indoor and outdoor levels of fine particulate mass, particle number concentrations and black smoke (BS) concluded that ambient concentrations of PM_2.5 _and BS can be used as good approximation of indoor concentrations.

Our panel of subjects consisted of male patients, with a history of heart disease, who were all on cardiac medication. Therefore, they represent a highly selected group and might have reacted differently compared to female patients or healthy persons. The chosen design attempted to balance the ability to study effects on the inflammatory system and to be representative for men with cardiovascular disease. A strength of our study is the achievement of 99% of all scheduled visits.

## Conclusion

The results for sCD40L add important new evidence to the hypothesis that higher levels of ambient air pollution lead to an inflammatory response in patients with CHD. Increased inflammation would increase the likelihood of serious arterial vascular thrombotic events in susceptible individuals such as patients with CHD, possibly explaining, in part, the observed association between air pollution and cardiovascular morbidity and mortality.

## Authors' contributions

RR worked on the acquisition of the data, performed the statistical analyses and drafted the manuscript;

RP performed the analyses of the sCD40L and has been involved in drafting the manuscript;

AS was substantially involved in the analyses of the data and revised the manuscript critically;

MF made substantial contribution to the interpretation of the data and the draft of the manuscript;

JC was involved in the acquisition of the air pollution data and was involved in the drafting of the manuscript;

GO made substantial contribution to the design of the study and reviewed the manuscript critically;

HEW was substantially involved in the design of the study and reviewed the manuscript critically;

AP was substantially involved in the design of the study, the data acquisition, the statistical analyses, the interpretation of the results and the drafting of the manuscript.

All authors have read and approved the final manuscript.

## References

[B1] Forastiere F, Stafoggia M, Picciotto S, Bellander T, D'Ippoliti D, Lanki T, von Klot S, Nyberg F, Paatero P, Peters A, Pekkanen J, Sunyer J, Perucci CA A Case-Crossover Analysis of Out-of-Hospital Coronary Deaths and Air Pollution in Rome, Italy. Am J Respir Crit Care Med.

[B2] von Klot S, Peters A, Aalto P, Bellander T, Berglind N, D'Ippoliti D, Elosua R, Hormann A, Kulmala M, Lanki T, Lowel H, Pekkanen J, Picciotto S, Sunyer J, Forastiere F Ambient air pollution is associated with increased risk of hospital cardiac readmissions of myocardial infarction survivors in five European cities. Circulation.

[B3] Peters A, Klot vS, Heier M, Trentinaglia I, Cyrys J, Hörmann A, Hauptmann M, Wichmann H-E, Löwel H (2005). Particulate Air Pollution and Nonfatal Cardiac Events.

[B4] Peters A, Pope CA Cardiopulmonary mortality and air pollution. Lancet.

[B5] Schwartz J (1999). Air pollution and hospital admissions for heart disease in eight U.S. counties. Epidemiology.

[B6] Donaldson K, Mills N, MacNee W, Robinson S, Newby D Role of inflammation in cardiopulmonary health effects of PM. Toxicol Appl Pharmacol.

[B7] Nemmar A, Nemery B, Hoet PH, Vermylen J, Hoylaerts MF Pulmonary inflammation and thrombogenicity caused by diesel particles in hamsters: role of histamine. Am J Respir Crit Care Med.

[B8] Silva VM, Corson N, Elder A, Oberdorster G (2005). The rat ear vein model for investigating in vivo thrombogenicity of ultrafine particles (UFP). Toxicol Sci.

[B9] Khandoga A, Stampfl A, Takenaka S, Schulz H, Radykewicz R, Kreyling W, Krombach F Ultrafine particles exert prothrombotic but not inflammatory effects on the hepatic microcirculation in healthy mice in vivo. Circulation.

[B10] Nemmar A, Hoylaerts MF, Hoet PH, Dinsdale D, Smith T, Xu H, Vermylen J, Nemery B Ultrafine particles affect experimental thrombosis in an in vivo hamster model. Am J Respir Crit Care Med.

[B11] Suwa T, Hogg JC, Quinlan KB, Ohgami A, Vincent R, van Eeden SF Particulate air pollution induces progression of atherosclerosis. J Am Coll Cardiol.

[B12] Ruckerl R, Ibald-Mulli A, Koenig W, Schneider A, Woelke G, Cyrys J, Heinrich J, Marder V, Frampton M, Wichmann HE, Peters A Air pollution and markers of inflammation and coagulation in patients with coronary heart disease. Am J Respir Crit Care Med.

[B13] Freedman JE CD40-CD40L and platelet function: beyond hemostasis. Circ Res.

[B14] Henn V, Slupsky JR, Grafe M, Anagnostopoulos I, Forster R, Muller-Berghaus G, Kroczek RA CD40 ligand on activated platelets triggers an inflammatory reaction of endothelial cells. Nature.

[B15] Phipps RP Atherosclerosis: the emerging role of inflammation and the CD40-CD40 ligand system. Proc Natl Acad Sci.

[B16] Kaufman J, Sime PJ, Phipps RP Expression of CD154 (CD40 ligand) by human lung fibroblasts: differential regulation by IFN-gamma and IL-13, and implications for fibrosis. J Immunol.

[B17] Akbiyik F, Ray DM, Gettings KF, Blumberg N, Francis CW, Phipps RP Human bone marrow megakaryocytes and platelets express PPARgamma, and PPARgamma agonists blunt platelet release of CD40 ligand and thromboxanes. Blood.

[B18] Ibald-Mulli A (2004). Effects of particulate air pollution on blood pressure and heart rate in subjects with cardiovascular disease: A multicentre approach. Environ Health Perspect.

[B19] Wichmann HE, Spix C, Tuch T, Woelke G, Peters A, Heinrich J, Kreyling WG, Heyder J (2000). Daily mortality and fine and ultrafine particles in Erfurt, Germany. Part I: Role of particle number and particle mass. Health Effects Institute Research Report.

[B20] Brand P, Gerhardt J, Below M, Georgi B, Heyder J (1992). Technical note: Performance of a mobile aerosol spectrometer for an in situ charcterisation of environmental aerosols in Frankfurt City. Atmospheric Environment.

[B21] Ramsay TO, Burnett RT, Krewski D (2003). The effect of concurvity in generalized additive models linking mortality to ambient particulate matter. Epidemiology.

[B22] Cyrys J, Dietrich G, Kreyling W, Tuch T, Heinrich J PM25 measurements in ambient aerosol: comparison between Harvard impactor (HI) and the tapered element oscillating microbalance (TEOM) system. Sci Total Environ.

[B23] Kingham S, Durand M, Aberkane T, Harrison J, Gaines Wilson J, Epton M (2006). Winter comparison of TEOM, MiniVol and DustTrak PM10 monitors in a woodsmoke environment. Atmospheric Environment.

[B24] Elzey BD, Tian J, Jensen RJ, Swanson AK, Lees JR, Lentz SR, Stein CS, Nieswandt B, Wang Y, Davidson BL, Ratliff TL (2003). Platelet-mediated modulation of adaptive immunity. A communication link between innate and adaptive immune compartments. Immunity.

[B25] Phipps RP, Kaufman J, Blumberg N Platelet derived CD154 (CD40 ligand) and febrile responses to transfusion. Lancet.

[B26] Blumberg N, Phipps RP, Kaufman J, Heal JM (2003). The causes and treatment of reactions to platelet transfusions. Transfusion.

[B27] Ahn ER, Lander G, Jy W, Bidot CJ, Jimenez JJ, Horstman LL, Ahn YS (2004). Differences of soluble CD40L in sera and plasma: Implications on CD40L assay as a marker of thrombotic risk. Thromb Res.

[B28] Andre P, Nannizzi-Alaimo L, Prasad SK, Phillips DR Platelet-derived CD40L: the switch-hitting player of cardiovascular disease. Circulation.

[B29] Geiser M Morphological aspects of particle uptake by lung phagocytes. Microsc Res Tech.

[B30] Nemmar A, Hoet PH, Vanquickenborne B, Dinsdale D, Thomeer M, Hoylaerts MF, Vanbilloen H, Mortelmans L, Nemery B Passage of inhaled particles into the blood circulation in humans. Circulation.

[B31] Mills NL, Amin N, Robinson SD, Anand A, Davies J, Patel D, de la Fuente JM, Cassee FR, Boon NA, MacNee W, Millar AM, Donaldson K, Newby DE Do inhaled carbon nanoparticles translocate directly into the circulation in humans?. Am J Respir Crit Care Med.

[B32] Meiring JJ, Borm PJ, Bagate K, Semmler M, Seitz J, Takenaka S, Kreyling WG The influence of hydrogen peroxide and histamine on lung permeability and translocation of iridium nanoparticles in the isolated perfused rat lung. Part Fibre Toxicol.

[B33] Berry JP, Arnoux B, Stanislas G, Galle P, Chretien J (1977). A microanalytic study of particles transport across the alveoli: role of blood platelets. Biomedicine.

[B34] Becker S, Soukup J Coarse(PM(2.5–10)), fine(PM(2.5)), and ultrafine air pollution particles induce/increase immune costimulatory receptors on human blood-derived monocytes but not on alveolar macrophages. J Toxicol Environ Health A.

[B35] Harding SA, Sarma J, Josephs DH, Cruden NL, Din JN, Twomey PJ, Fox KA, Newby DE Upregulation of the CD40/CD40 ligand dyad and platelet-monocyte aggregation in cigarette smokers. Circulation.

[B36] Tan WC, Qiu D, Liam BL, Ng TP, Lee SH, van Eeden SF, D'Yachkova Y, Hogg JC (2000). The human bone marrow response to acute air pollution caused by forest fires. Am J Respir Crit Care Med.

[B37] Schwartz J (2001). Air pollution and blood markers of cardiovascular risk. Environ.

[B38] Salvi S, Blomberg A, Rudell B, Kelly F, Sandstrom T, Holgate ST, Frew A (1999). Acute inflammatory responses in the airways and peripheral blood after short-term exposure to diesel exhaust in healthy human volunteers. Am J Respir Crit Care Med.

[B39] Ghio AJ, Hall A, Bassett MA, Cascio WE, Devlin RB (2003). Exposure to concentrated ambient air particles alters hematologic indices in humans. Inhalation Toxicology.

[B40] Clarke RW, Coull B, Reinisch U, Catalano P, Killingsworth CR, Koutrakis P, Kavouras I, Murthy GG, Lawrence J, Lovett E, Wolfson JM, Verrier RL, Godleski JJ (2000). Inhaled concentrated ambient particles are associated with hematologic and bronchoalveolar lavage changes in canines. Environ Health Perspect.

[B41] Seaton A, Soutar A, Crawford V, Elton R, McNerlan S, Cherrie J, Watt M, Agius R, Stout R (1999). Particulate air pollution and the blood. Thorax.

[B42] Inwald DP, McDowall A, Peters MJ, Callard RE, Klein NJ CD40 is constitutively expressed on platelets and provides a novel mechanism for platelet activation. Circ Res.

[B43] Kaufman J, Graf BA, Leung EC, Pollock SJ, Koumas L, Reddy SY, Blieden TM, Smith TJ, Phipps RP (2001). Fibroblasts as sentinel cells: role of the CDcd40-CDcd40 ligand system in fibroblast activation and lung inflammation and fibrosis. Chest.

[B44] Sempowski GD, Chess PR, Phipps RP CD40 is a functional activation antigen and B7-independent T cell costimulatory molecule on normal human lung fibroblasts. J Immunol.

[B45] Marder VJ, Rosove MH, Minning DM (2004). Foundation and sites of action of antithrombotic agents. Best Practice and Research Clinical Haematology.

[B46] van Eeden SF, Tan WC, Suwa T, Mukae H, Terashima T, Fujii T, Qui D, Vincent R, Hogg JC Cytokines involved in the systemic inflammatory response induced by exposure to particulate matter air pollutants (PM(10)). Am J Respir Crit Care Med.

[B47] Rubin E, Farber JL (1998). Pathology.

[B48] von Andrian UH, Mackay CR Advances in immunology: T-cell function and migration – Two sides of the same coin. N Engl J Med.

[B49] Luster AD Chemokines – Chemotactic cytokines that mediate inflammation. N Engl J Med.

[B50] Ghio AJ, Kim C, Devlin RB (2000). Concentrated ambient air particles induce mild pulmonary inflammation in healthy human volunteers. Am J Respir Crit Care Med.

[B51] Pietropaoli AP, Frampton MW, Hyde RW, Morrow PE, Oberdorster G, Cox C, Speers DM, Frasier LM, Chalupa DC, Huang LS, Utell MJ (2004). Pulmonary function, diffusing capacity, and inflammation in healthy and asthmatic subjects exposed to ultrafine particles. Inhal Toxicol.

[B52] Frampton MW, Stewart JC, Oberdorster G, Morrow PE, Chalupa D, Pietropaoli AP, Frasier LM, Speers DM, Cox C, Huang LS, Utell MJ (2006). Inhalation of ultrafine particles alters blood leukocyte expression of adhesion molecules in humans. Environ Health Perspect.

[B53] Batalha JR, Saldiva PH, Clarke RW, Coull BA, Stearns RC, Lawrence J, Murthy GG, Koutrakis P, Godleski JJ (2002). Concentrated ambient air particles induce vasoconstriction of small pulmonary arteries in rats. Environ Health Perspect.

[B54] Peters A, Döring A, Wichmann HE, Koenig W (1997). Increased plasma viscosity during air pollution episode: A link to mortality?. Lancet.

[B55] Cyrys J, Heinrich J, Brauer M, Wichmann HE (1998). Spatial variability of acidic aerosols, sulfate and PM10 in Erfurt, Eastern Germany. Journal of Exposure Analysis and Environmental Epidemiology.

[B56] Janssen NAH, Hoek G, Brunekreef B, Harssema H, Mensink I, Zuidhoh A (1998). Personal Sampling of Particles in Adults: Relation among Personal, Indoor, and Outdoor Air Concentrations. Am J Epidemiol.

[B57] Ebelt ST, Wilson WE, Brauer M (2005). Exposure to ambient and nonambient components of particulate matter: a comparison of health effects. Epidemiology.

[B58] Cyrys J, Pitz M, Bischof W, Wichmann HE, Heinrich J (2004). Relationship between indoor and outdoor levels of fine particle mass, particle number concentrations and black smoke under different ventilation conditions. J Expo Anal Environ Epidemiol.

